# Experimental Investigation of Damping Properties of Selected Polymer Materials

**DOI:** 10.3390/ma17123021

**Published:** 2024-06-20

**Authors:** Lucjan Witek, Piotr Łabuński

**Affiliations:** 1 Department of Aerospace Engineering, Rzeszow University of Technology, al. Powst. Warszawy 8, 35-959 Rzeszów, Poland; 2 Doctoral School of Engineering and Technical Sciences, Rzeszow University of Technology, al. Powst. Warszawy 12, 35-959 Rzeszów, Poland; d506@stud.prz.edu.pl

**Keywords:** viscoelastic materials, passive vibration isolation, experimental modal analysis, free layer damping (FLD), constrained layer damping (CLD)

## Abstract

This paper presents the results of an experimental modal analysis of a beam covered by polymer materials used as a passive vibration isolation. The main aim of this study was to determine the damping properties of selected viscoelastic materials. In order to check the damping properties of tested materials, an experimental modal analysis, with the use of an electrodynamic vibration system, was performed. In this study, four kinds of specimens were considered. In the first step of the work, the beam made out of aluminum alloy was investigated. Afterwards, a cantilever beam was covered with a layer of bitumen-based material acting as a damper. This method is commonly known as a free layer damping treatment (FLD). In order to increase the damping capabilities, the previous configuration was improved by fixing a thin aluminum layer directly to the viscoelastic core. Such a treatment is called constrained layer damping (CLD). Subsequently, another polymer (butyl rubber) in the CLD configuration was tested for its damping properties. As a result of the performed experimental modal analysis, the frequencies of resonant vibrations and their corresponding amplitudes were obtained. The experimental results were used to quantitatively evaluate the damping properties of tested materials.

## 1. Introduction

Vibrations occurring in many mechanical structures are an unfavorable phenomenon. In many cases, vibrations are accompanied by cyclic changes in stress, which contributes to the reduction of the fatigue life of the structure. For example, a blade of a compressor that is subjected to resonant vibrations can many times decrease the fatigue life of turbine engine [1,2]. The vibration problem is common in thin-walled structures that are widely used in the aerospace and automotive industries. The small thickness of the structural elements results in their low bending stiffness, which favors the formation of high-amplitude vibrations. When the excitation frequency (most often associated with vibrations of a turbine or piston engine, as well as with aerodynamic vibrations) coincides with the natural frequency, the structure falls into resonance.

The resonance of structure creates an acoustic wave in the cabin of an airplane or car and reduces the comfort of traveling. Resonant vibrations of the cabin cause noise and, consequently, accelerated fatigue of car drivers and aircraft pilots, indirectly affecting transport safety.

One of the methods of reducing the vibrations of thin-walled structures is active vibration isolation. This method is based on the active (on-line) monitoring of structure vibrations and generation by the electronic system and miniature vibrators in the structure of the opposite phase. The disadvantage of active vibration isolation is the need to use complex electronic systems that require continuous power supply and the high cost of such installation.

**Figure 1:**
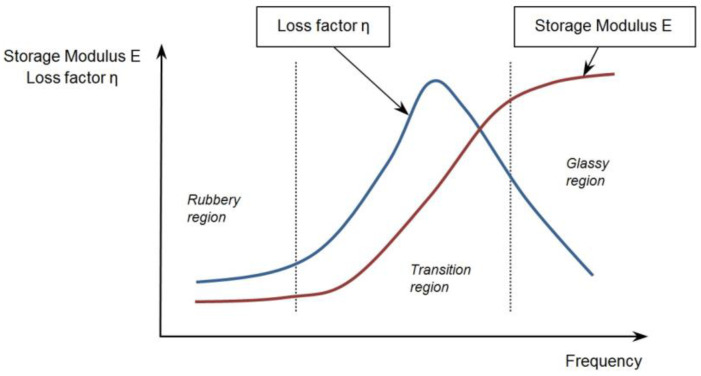
Loss factor, η, and storage modulus, E, as a function of frequency [6,7].

An alternative method of damping vibrations is passive vibration isolation. In this method, special damping mats are glued to the surface of the element. Viscoelastic (polymer) materials are most often used in the actual vibration isolation technique, as they effectively reduce the vibration amplitude of thin-walled structures and reduce the noise. Passive damping has many advantages. It does not need external power, and such dampers are less expensive than active equivalents. As an economical solution, viscoelastic damping materials are applied in over 85% of the passive damping treatments [3]. The most widely applied polymers are bitumen and butyl-based material [4]. Such materials have both viscous and elastic properties. Thus, after removing the load from them, some of the energy is dissipated in the form of thermal energy, and some is recovered. Their damping capabilities strongly vary with the frequency and temperature (Figure 1). The storage modulus, E, has the highest value in the glassy region. The damping effect could be expressed as the loss factor and has the highest value in the transition region (Figure 1). The disadvantage of viscoelastic materials is their susceptibility to temperature [5].

**Figure 2:**
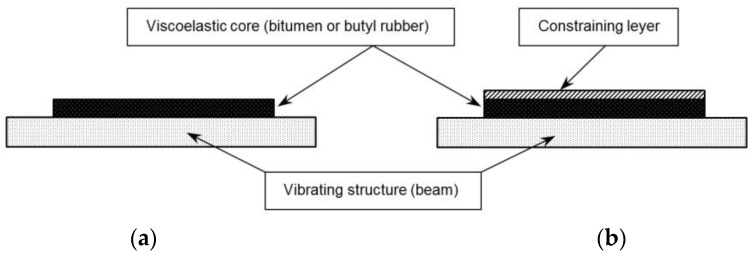
Viscoelastic materials damping: (**a**) FLD treatment and (**b**) CLD treatment [6,7].

In order to achieve a significant damping performance, a vibrating structure is often covered with a layer of viscoelastic polymer. Substantially, there are two approaches applied: free layer damping (FLD, Figure 2a) and constrained layer damping (CLD, Figure 2b) [8,9,10].

In the first configuration (FLD), a viscoelastic layer is attached to a vibrating structure. In this case, energy is dissipated through extension. The second treatment (CLD) is basically FLD with an additional metallic layer. Thanks to the constraining layer, energy dissipates through shear deformation [6]. One of the first people who observed the increasing damping performance thanks to the additional layer was Kerwin [11,12]. In [13], the mathematical formula determining the transverse displacement of a three-layer beam with a viscoelastic core was described by Mead and Markus. The formula for the eigenfrequency in a multi-layer beam was described by Rao in [14]. Hujare and Sahasrabudhe performed an experiment in which the damping properties of seven different viscoelastic polymers (CLD treatment) were examined [15]. In this paper, the aluminum beam (covered by damping materials) was excited by a modal hammer. The dampened structure was tested in the frequency range from 0 to 600 Hz. To estimate the damping factor, a half-power bandwidth method was used.

In Reference [16], a viscoelastic damping material (water-based acrylic acid, 0.8 mm thick) was used in the modal experiments of a beam made of different materials (stainless steel, composite). In this work, a frequency response function in the range of 0–1600 Hz was obtained for seven measure points. The results of an experimental modal analysis performed to evaluate the frequency response function of a rectangular beam were presented in Reference [17]. As a damped material, polymer tape (0.14 mm thick, width of 50 mm) was used. The tests were performed on three aluminum alloy plates with CLD layers applied in varying layouts: parallel to the long axis of the plate, perpendicular to the long axis and 45° to the long axis. In the experiment, the mini-shaker and a single pointer Laser Doppler vibrometer were used. As the main result, the frequency response function was obtained for the investigated plates in the frequency range of 0–400 Hz.

The aim of FLD and CLD treatments is to decrease the vibration amplitude and, hence, to minimize noise and also the stress in order to extend the fatigue life of structures. The results of an experimental vibration test of the steel beam (important from the point of view of automotive industry) damped by viscoelastic materials in the FLD configuration were published in Reference [18]. Due to the fact that the aluminum alloys are commonly used in thin-walled aircraft structures, there was a need to conduct an experimental modal analysis (in extended approach both FLD and CLD) for aluminum alloy elements.

The main goal of this work was the experimental determination of the effectiveness of passive vibration isolation in the FLD and CFD configuration of samples made of aluminum alloy, damped with a polymer material (bituminous and butyl rubber). To assess the effectiveness of vibration isolation, the vibration amplitude reduction index (relative amplitude) was proposed, which is important, as the fatigue life of the structure could be considered as a continuation of this work. The results of the presented research are important not only from the scientific but also from the practical point of view and may be useful in the aviation industry (in the design process to reduce noise in the passenger cabin of an airplane and also to increase the structure’s fatigue life).

## 2. Materials and Methods

**Figure 3:**
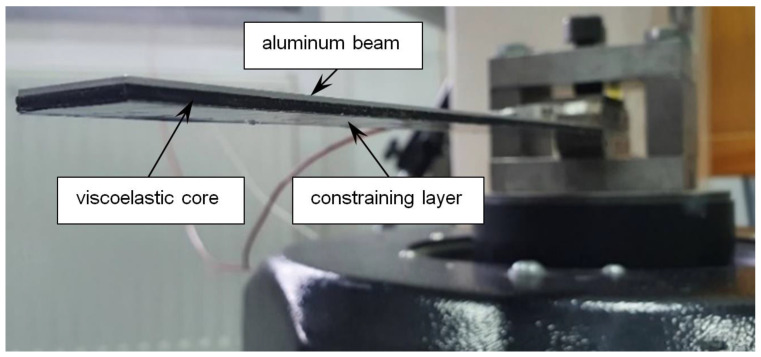
View of investigated beam fixed to the head of the shaker in CLD treatment.

In order to determine the damping capabilities of the tested polymer materials, an experimental modal analysis was performed. For this purpose, the Unholtz-Dickie UDCO TA250 electrodynamic vibration system was used. This system consisted of a computer, controller, amplifier and shaker. In this investigation, the PCB sensor was used for the modal analysis. During the experiment, several configurations of the beam were investigated. In Figure 3, the specimen with CLD treatment fixed to the movable head of the shaker is shown.

In order to determine the damping performance of investigated viscoelastic materials, the Oberst beam method was used [6]. The cantilever beam was made out of aluminum alloy AW-2017A [19,20]. The dimensions of the beam were as follows: 300 mm (length), 20 mm (width) and 1 mm (thickness).

**Figure 4:**
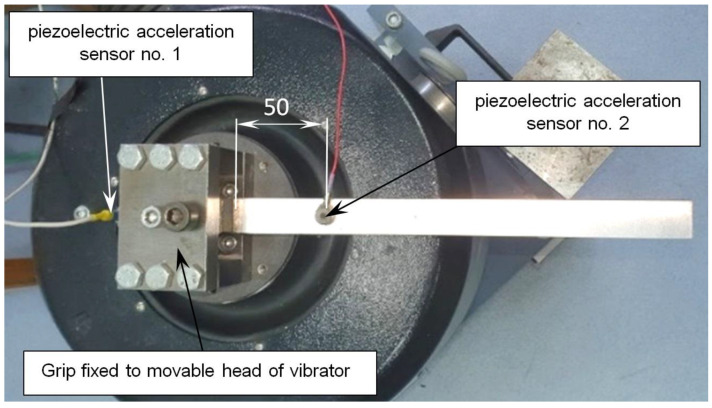
Location of piezoelectric acceleration sensors used in modal analysis (sensor no. 1 defined in control channel; and sensor no. 2—measure channel).

The location of the piezoelectric vibration sensors (ICP type) used in modal analysis is presented in Figure 4. Sensor no. 1 was used to control the acceleration (*A_1_*) of the shaker head. The sensitivity of this sensor was equal to 94 mV/g. The signal from this sensor was assigned to the control channel. Sensor no. 2 was used to measure the beam acceleration (*A_2_*). The signal from sensor no. 2 was assigned to measure the channel. The sensitivity of sensor no. 2 equals 10.04 mV/g. The mass of both sensors equals 2 g.

**Figure 5:**
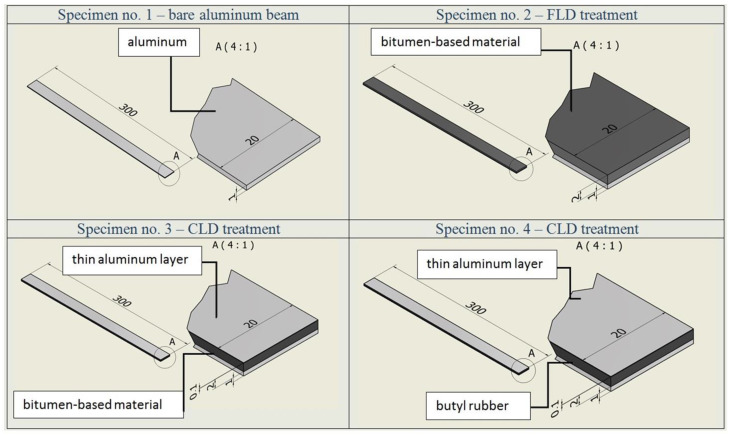
Specimens used in experimental investigations.

The thickness of the tested viscoelastic materials (bitumen-based and butyl rubber) was 2 mm (Figure 5). The density of the bituminous material used to dampen vibrations (in specimen nos. 2 and 3) was 1.8 kg/dm^3^. The storage modulus of viscoelastic materials depends on temperature and frequency. The storage modulus, E’, of investigated bitumen material (measured in temperature of 20 °C) oscillates between 55 and 600 MPa (in the frequency range of 10–3000 Hz). The density of butyl rubber used in specimen no. 4 was 2 kg/dm^3^. The storage modulus, E’, of investigated butyl rubber (in the range of 10–2500 Hz at temperature of 20 °C) was a value from 120 MPa to 230 MPa. At a higher frequency (2500–3700 Hz), the storage modulus of the butyl rubber increases from 230 MPa to the value of about 670 MPa. The abovementioned storage modules were experimentally determined by the authors of this paper as a part of separate studies.

In the presented investigations, the mats containing a self-adhesive layer were used. During sample preparation, the mat with the adhesive layer was pressed to the beam surface and then rolled. The high local pressure obtained in this way allowed for a strong connection between the mat and the beam. The additional thin aluminum layer (in CLD configuration) was connected to the damping mat using the polymer glue.

In the experimental analysis, four configurations of the investigated beam were examined. In the description of the experimental research presented in this work, the terms “specimen” or “sample” are used interchangeably. The dimensions of the specimens presented in Figure 5 are defined in millimeters. In the first step, the aluminum beam without damping material was analyzed (specimen no. 1, Figure 5). Afterwards, the cantilever beam was covered with a layer of bitumen-based material (specimen no. 2, FLD treatment). In order to increase the damping capabilities, the previous configuration was improved by fixing a thin aluminum layer directly to the viscoelastic core (specimen no. 3, CLD treatment). In the last configuration (specimen no. 4), a layer of butyl rubber, constrained by an aluminum plate, was attached to the cantilever beam (CLD treatment).

The thickness of both aluminum constraining (additional) layers used in specimen nos. 3 and 4 was 0.1 mm. All samples were tested at room temperature (20 °C). The specimens were prepared according to ASTM Standard E756(05) [21]. The experimental modal analysis was performed in the high frequency range, from 10 Hz to 4 kHz. The frequency sweep rate was set as linear. The amplitude of vibration was measured on-line via a piezoelectric accelerometer on the beam (channel no 2) and on the shaker head (channel no 1). In the vibration test, the constant intensity of acceleration of 1 g was defined for the shaker head (where 1 g = 9.81 m/s^2^). During the experimental tests, the frequency (of shaker head, grip and the investigated beam) was slowly increased.

## 3. Results

**Figure 6:**
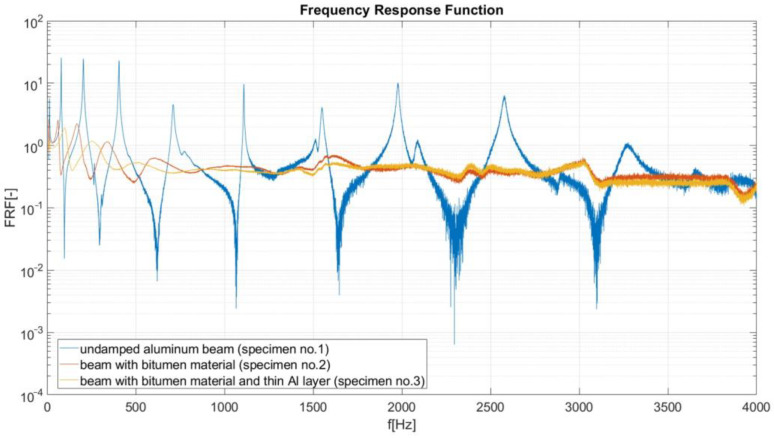
Frequency response curves for specimen numbers 1, 2 and 3.

**Figure 7:**
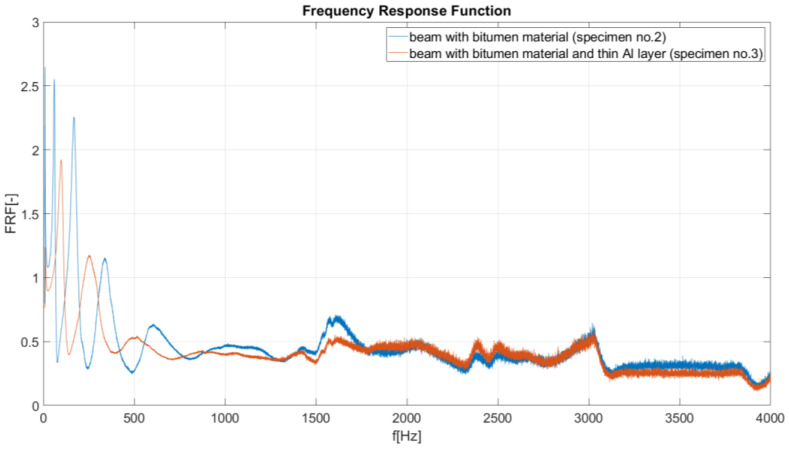
Frequency response curves for specimens: no. 2 (FLD treatment) and no. 3 (CLD treatment).

**Figure 8:**
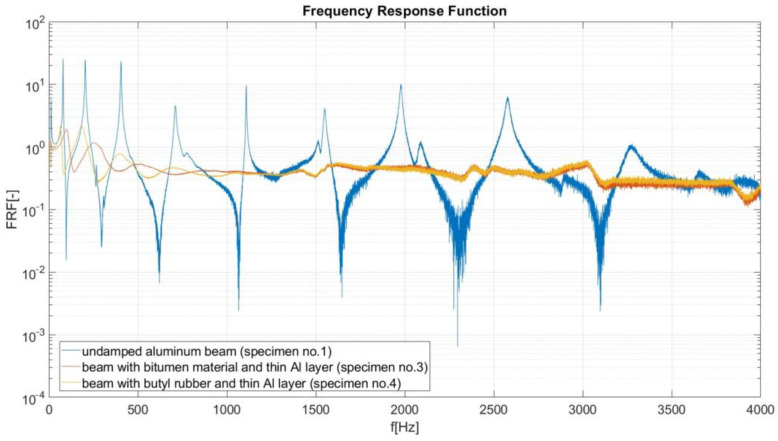
Frequency response curves for specimens nos. 1, 3 and 4.

**Figure 9:**
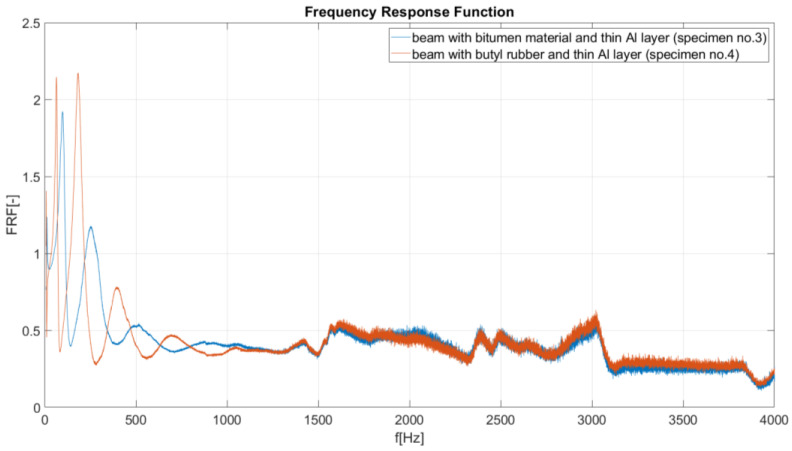
Frequency response curves for specimen nos. 3 and 4.

In this investigation, the LMS data-acquisition controller and software were used. During the modal analysis, the excitation frequency increased at a rate of 1 Hz/s, and the LMS system recorded the object response (beam vibration amplitude) every 0.1 Hz. Based on the results of performed experimental analysis, the amplitude–frequency characteristics were created for considered specimens. On the vertical axis of these characteristics, the Frequency Response Function (FRF) is defined as a function used to quantify the response of the beam to an excitation, normalized by the magnitude of this excitation. During the FRF calculation, the signal from channel no. 2 (acceleration A_2_ measured on the beam) was divided by the signal from reference channel no. 1 (acceleration A_1_ of shaker head). The reduction in the value of the FRF function (in peaks, recognized as resonance (Figure 6, Figure 7, Figure 8 and Figure 9) is useful when performing a preliminary assessment of the effectiveness of the passive vibration isolation of the analyzed samples. In the description of the results, the vibration modes are correlated with the resonance frequencies of the undamped beam (sample no. 1).

Due to the availability of results (11 resonant frequencies) and the inclusion of various damping methods (FLD and CLD), as well as various damping materials, the results of the experimental tests were divided into four groups:

I. FRF comparison for an undamped beam (specimen no. 1), a damped beam (specimen no. 2, bitumen material, FLD configuration) and a damped beam (specimen no. 3, bitumen with additional thin aluminum layer, CLD configuration, Figure 6). The reduction in the FRF value (at each resonance) in the dumped beam is an indicator of the effectiveness of passive vibration isolation. The results presented in Figure 6 show that the greatest effectiveness of passive vibration isolation using a bituminous mat (in FLD and CLD solutions) occurs in the frequency range from about 500 Hz to 3500 Hz. In the range of first resonant frequencies (10–500 Hz), a slightly smaller vibration dumping is observed. Due to the wide range of results presented in Figure 6, it is not possible to compare the effectiveness of passive vibration isolation in the FLD and CLD variants.

II. FRF comparison for a damped beam (specimen no. 2, bitumen material, FLD treatment) and a damped beam (specimen no. 3, bitumen with an additional aluminum layer, CLD treatment, Figure 7). As seen in Figure 7, in the range of about 2000–4000 Hz, the damping properties of the two methods (FLD and CLD) are almost equal. For the first considered vibration modes (from 1st to 5th), the CLD treatment provides better damping. Moreover, the 2nd mode of vibration does not occur in specimen no. 3.

III. FRF comparison for an undamped beam (specimen no. 1), a damped beam (sample no. 3, bitumen material, CLD treatment) and a damped beam (sample no. 4, butyl rubber, CLD treatment, Figure 8). As seen in Figure 8, in the frequency range up to 1000 Hz, the moderate damping of vibrations is observed for both specimens nos. 3 and 4. In the frequency range of 1000–4000 Hz, a large damping is observed for both samples. In the high frequency range, it is difficult to assess which type of damping material (butyl rubber or bitumen) is more effective in damping vibrations (Figure 8).

IV. FRF comparison for a damped beam (specimen no. 3, bitumen, CLD) and a damped beam (sample no. 4, butyl rubber, CLD (Figure 9). The results presented in Figure 9 show that, in the frequency range 10–1000 Hz, the bitumen-based material has better damping properties than the butyl rubber. In the higher frequency range (1000–4000 Hz), both of specimens have similar amplitudes, meaning that both materials in the CLD configuration (butyl rubber and bitumen material) have similar damping properties.

## 4. Discussion

Due to the large number of result configurations, the vibration amplitude defined by a double index is introduced. For example, subscript 2 in vibration amplitude $A_{2}^{3}$ means that the acceleration was measured on the beam surface (sensor no. 2, Figure 4). Superscript 3 in the expression $A_{2}^{3}$ means that the amplitude value measured on the beam applies to sample no. 3. In general, the superscript refers to the sample number, and the subscript refers to the measurement location (measurement channel number). In order to calculate the relative amplitudes, the amplitudes $A_{2}^{2}$, $A_{2}^{3}$ and $A_{2}^{4}$ of the damped beam (obtained for specimens nos. 2, 3 and 4) were divided by the amplitude of undamped beam $A_{2}^{1}$ (specimen 1). All amplitudes mentioned in this section were measured on the beam surface (by piezoelectric acceleration sensor no. 2, Figure 4).

Based on described-below procedure, the relative amplitudes ($A_{2}^{2}/A_{2}^{1}$, $A_{2}^{3}/A_{2}^{1}$, $A_{2}^{4}/A_{2}^{1}$) were computed for each resonance for specimens 2, 3 and 4. The quantitative results of performed modal analysis are presented in Table 1. The smallest values of the relative amplitude of each resonant vibration mode for samples nos. 2, 3 and 4 are shown in bold (Table 1). Relative amplitude is an important parameter in assessing the reduction in vibrations of mechanical objects and can be used to evaluate the effectiveness of passive vibration isolation. The value of relative amplitude is also important because of the fact that reduction in amplitude causes a decrease both in the displacement (deformation) and the stress in the damped object. A reduction in stress causes an increase of the fatigue life of a structure.

**Figure 10:**
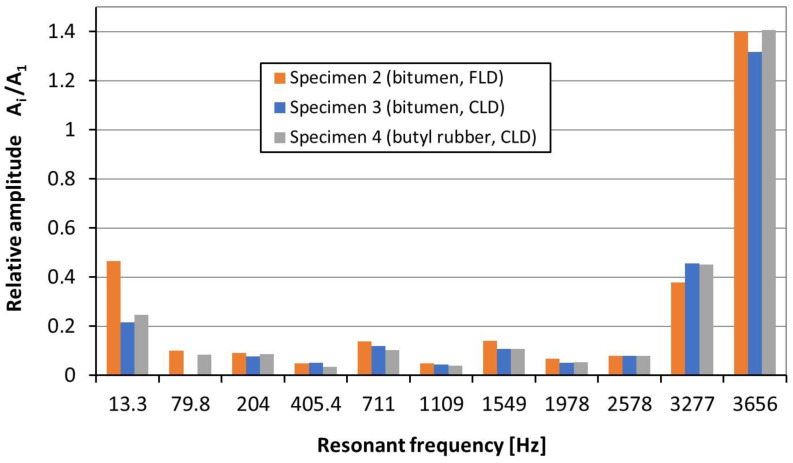
Relative amplitude of acceleration, $A_{2}^{i}/A_{2}^{1}$, of specimens nos. 2, 3 and 4 for all resonant frequencies.

**Figure 11:**
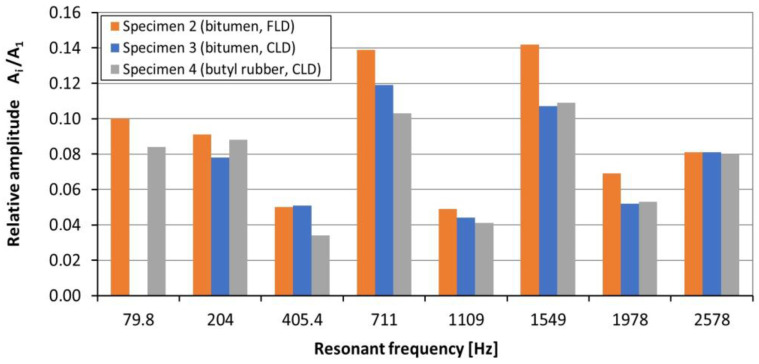
Relative amplitude of acceleration, $A_{2}^{i}/A_{2}^{1}$, of specimens nos. 2, 3 and 4 for resonant frequencies range of 79.8–2578 Hz.

Based on the research results (Table 1), it can be generally concluded that the CLD treatment has better damping capabilities than FLD. Due to the large number of results, the values of the relative vibration amplitude, $A_{2}^{i}/A_{2}^{1}$ (where i = 2, 3, 4), are presented in bar charts (Figure 10 and Figure 11).

The obtained results of the performed investigation showed that, for low resonant frequencies (13.3 Hz, 79.8 Hz and 204 Hz, modes nos. 1–3), the smallest relative amplitude (0.078) was recorded for specimen 3 (Figure 10 and Table 1). The above result means that the amplitude of the vibrations (accelerations) of the damped beam (sample no. 3, bitumen, CLD) was reduced by almost 13 times (compared to the undamped beam (sample no. 1). As seen from Figure 10, the bitumen in CLD configuration for low frequencies (13.3 Hz and 204 Hz) has somewhat better damping properties (in terms of amplitude reduction) than bitumen in the FLD treatment. In the case of mode 10 (resonant frequency of 3277 Hz), the acceleration amplitude was reduced from 1 (for an undamped beam) to a value of approximately 0.4. For mode 11 (3656 Hz), the vibration amplitude after the use of the damping material increased by approximately 40%.

The test results indicate that the greatest vibration reduction occurred in the frequency range of 79.8–2578 Hz. In the mentioned range, the relative amplitude was from 0.034 to 0.142 (Table 1). The results presented in Figure 11 show that, in a frequency range from 405.4 Hz to 1109 Hz (vibration modes nos. 4, 5 and 6), the applications of butyl rubber in CLD configuration enables the greatest reduction in the amplitude of resonant vibration. At a higher frequency (1549 Hz and 1978 Hz), the highest effectiveness in damping vibrations occurs for specimen 3 (bitumen, CLD). It can be seen that, for the frequencies of 1549 Hz, 1978 Hz and 2578 Hz, the differences in the relative amplitude of the bituminous material and the butyl rubber (both in CLD configuration) are very small.

## 5. Conclusions

In this study, the experimental modal analysis of cantilever beams with viscoelastic damping was performed. In the presented analysis, four configurations of specimens were considered (a bare aluminum cantilever beam, a beam covered with layer of bitumen-based material (FLD), and sandwich structures (CLD) with bitumen and butyl rubber as a core). As the result of the performed modal analysis, the frequencies of resonant vibrations and their corresponding amplitudes were obtained. In order to assess the effectiveness of damping materials, the relative amplitude of vibrations was introduced.

The results of experimental investigations showed that the best effectiveness of the tested damping materials occurs in the frequency range of 79.8–2578 Hz. In this frequency range, the relative amplitude of the aluminum beam dumped by butyl rubber (in CLD configuration) was decreased from 1 (undamped beam) to the value of about 0.034–0.109 (the amplitude was, in this case, reduced by a factor of 9 to 29). The bituminous material in the CLD configuration (in the range of 79.8–2578 Hz) also has very good damping properties, where the relative amplitude value is from 0.051 to 0.119. For comparison, the relative amplitude of the beam covered with bituminous material (in the FLD configuration) is from 0.049 to 0.142.

The greatest difference in the damping intensity of the bituminous material (between the FLD and CLD configurations) occurs at a low resonant frequency, i.e., 13.3 Hz (for specimen no. 2 (FLD), the relative amplitude equals 0.465, and for the CLD variant (specimen no. 3), 0.217). The test results indicated that moderate damping (for samples 2–4) occurred for two separated frequencies (13.3 Hz and 3277 Hz). For the abovementioned resonant frequencies, the relative amplitudes were adequately 0.217 and 0.456. Additionally, it can be seen that, for the 11th resonant frequency (3656 Hz), the relative vibration amplitude was approximately 1.4, meaning that, after adding the damping material to the beam, the unfavorable phenomenon of increasing the vibration amplitude occurred.

The obtained results concern one specific example of the mechanical structure under specific boundary conditions. It should therefore be stated that, in structures with a greater degree of geometric complexity, the reduction of the vibration amplitude may have different values. In the future, the authors of this publication intend to test the effectiveness of passive vibration isolation of the fuselage panel of a turboprop aircraft, which is excited by the acoustic wave created by the rotating propeller. In this work, we planned to use bituminous and butyl material to reduce the vibration amplitude of the aircraft skin. Moreover, the impact of passive vibration isolation on the increase in the fatigue life of the fuselage will be examined in the future.

Due to planned further tasks related to aviation structures, the authors of this work focused on quantitative research on passive vibration isolation of a thin-walled structure with low bending stiffness, made of an aluminum alloy used in aviation. For this reason, the relative amplitude (important also from the fatigue point of view) was selected as a key indicator in assessing the effectiveness of passive vibration isolation.

The presented results are significant from both the research and the practical point of view. Reducing the vibration amplitude of structures is beneficial for two reasons. Firstly, it reduces noise in the vehicle or aircraft cabin, which increases not only comfort but also affects crew fatigue and, indirectly, transport safety [22,23,24]. From the point of view of material mechanics, large vibration amplitudes cause stress pulsation, which reduces the fatigue life of a structure. Better knowledge about the damping properties of viscoelastic materials may be useful in the aviation and automotive industries to design more comfortable and durable aircraft and motor vehicles.

## Figures and Tables

**Table 1:** Frequency of resonant vibration, amplitude of acceleration (absolute), and relative amplitude of acceleration of specimens.

Specimen 1 (Beam without Damping)			Specimen 2 (Bitumen-Based Material FLD)			Specimen 3 (Bitumen-Based Material CLD)			Specimen 4 (Butyl Rubber CLD)		
No. of Mode	Res. Freq. [Hz]	Ampl. of Accel. $A_{2}^{1}$ [g]	Res. Freq. [Hz]	Ampl. of Accel. $A_{2}^{2}$ [g]	Rel. Ampl. $A_{2}^{2}/A_{2}^{1}$	Res. Freq. [Hz]	Ampl. of Accel. $A_{2}^{3}$ [g]	Rel. Ampl. $A_{2}^{3}/A_{2}^{1}$	Res. Freq. [Hz]	Ampl. of Accel. $A_{2}^{4}$ [g]	Rel. Ampl. $A_{2}^{4}/A_{2}^{1}$
**1**	13.3	5.689	9.7	2.647	0.465	12.1	1.232	**0.217**	9.4	1.406	0.247
**2**	79.8	25.550	61.1	2.551	0.100	-	-	-	65	2.145	0.084
**3**	204	24.710	168.8	2.258	0.091	97.6	1.923	**0.078**	183.1	2.174	0.088
**4**	405.4	23.080	338.9	1.155	0.050	252.5	1.177	0.051	395.7	0.782	**0.034**
**5**	711	4.587	606.6	0.638	0.139	516.1	0.547	0.119	697.2	0.473	**0.103**
**6**	1109	9.696	1042	0.477	0.049	941.5	0.424	0.044	1050	0.396	**0.041**
**7**	1549	4.114	1542	0.584	0.142	1533	0.441	**0.107**	1533	0.448	0.109
**8**	1978	10.170	1578	0.702	0.069	1568	0.534	**0.052**	1570	0.541	0.053
**9**	2578	6.402	2063	0.519	0.081	2056	0.518	0.081	2043	0.511	**0.080**
**10**	3277	1.134	2513	0.428	**0.378**	2507	0.517	0.456	2503	0.511	0.451
**11**	3656	0.450	3029	0.629	1.399	3013	0.593	**1.318**	3021	0.633	1.406

## Data Availability

The original contributions presented in the study are included in the article, further inquiries can be directed to the corresponding author.
